# Chronic Diseases, Lack of Medications, and Depression Among Syrian Refugees in Jordan, 2013–2014

**DOI:** 10.5888/pcd12.140424

**Published:** 2015-01-29

**Authors:** Omar Salem Gammouh, Ahmed Mohammad Al-Smadi, Loai Issa Tawalbeh, Laurice Sami Khoury

**Affiliations:** Author Affiliations: Ahmed Mohammad Al-Smadi, American University of Madaba, Madaba, Jordan; Loai Issa Tawalbeh, AL-ALBayt University, Mafraq, Jordan; Laurice Sami Khoury, Caritas Jordan, Amman, Jordan.

## Abstract

**Introduction:**

Studying mental and physical health problems in refugees facilitates providing suitable health care, thus improving their quality of life. We studied depression tendency in Syrian refugees in Jordan in the light of chronic diseases and medication availability. Also, depression prevalence and depression comorbidity with chronic diseases were identified.

**Methods:**

In this multicenter cross-sectional survey, data from Syrian refugees attending Caritas centers in 6 Jordanian cities from November 2013 through June 2014 were analyzed. Participants’ demographics, depression, previously diagnosed chronic diseases, and newly diagnosed chronic diseases and the availability of medications were studied. Logistic regression was used to examine predictors for depression.

**Results:**

Of 765 refugees who participated, about one-third demonstrated significant depression as measured by the Beck Depression Inventory. Descriptive analyses showed that depression was comorbid in 35% of participants with previously diagnosed chronic diseases and in 40% of participants with newly diagnosed chronic diseases. Newly diagnosed chronic diseases and lack of medications significantly contributed to depression, but the regression model as a whole explained less than 5% of the variance.

**Conclusion:**

Because the regression model showed low effect size, we concluded that newly diagnosed chronic diseases and medication shortages could not predict depression in Syrian refugees residing in Jordan. Therefore, further studies of additional factors are recommended. Prompt measures have to be taken to prevent the spread of chronic diseases and improve mental health in this fragile population.

## Introduction

Since the beginning of the Syrian conflict in 2011, Syrians have been fleeing to neighboring countries. Jordan, which is to the south of Syria and shares Syria’s southern border, is hosting Syrian refugees daily. The number of officially assisted Syrian refugees has exceeded 1.4 million (1).

Refugees’ physical and mental health represents a medical challenge. Compared with undisplaced populations, refugees have higher prevalences of chronic diseases because of competition for basic life needs and lack of medications and appropriate health care (2). Chronic diseases, whether diagnosed before migration or newly diagnosed after migration, require special attention and monitoring because of their impact on mental health and normal individual functioning (3). Controlling chronic diseases requires adequate numbers of pharmacists and medications (4).

Depression may affect physical health problems, particularly chronic diseases (5). Traumatized Cambodian and Southeast Asian adults in the United States reported having higher rates of diabetes and cardiovascular disease compared with the general population (6; Khmer Health Associates, unpublished data, 2003).

Understanding the connection between depression and chronic diseases is of utmost importance to improving health care for communities (7), especially in vulnerable groups such as refugees who are already at higher risk for developing both depression and chronic diseases (8). We hypothesized that depression in Syrian refugees is predicted by previously diagnosed chronic diseases, newly diagnosed chronic diseases, and shortage of medications for chronic and nonchronic diseases. To our knowledge, there are no published data related to depression prevalence, chronic diseases, and medication availability among Syrian refugees in Jordan. Our study’s primary objective was to examine whether previously diagnosed chronic diseases, chronic diseases newly diagnosed in Jordan, and medication shortages can predict depression in a cohort of Syrian refugees residing in Jordan. Secondary objectives were to determine depression prevalence and comorbidity with chronic diseases among Syrian refugees in Jordan.

## Methods

A cross-sectional study was conducted to meet the study objectives. The cross-sectional method was selected as it is the most appropriate method to explore the prevalence of depression among the study participants and to examine the predictors for depression among participants.

Syrian refugees live in overcrowded houses, and adults work in labor to support their families. Several nongovernment organizations support them financially to pay for rent and basic life needs. We used the convenience sample technique in recruiting the participants. Sample size was calculated based on a confidence level of 95%, confidence interval of 5%, and an estimated population size of 1 million. The results indicated the need for 385 or more participants. As a result, we decided to include as many participants as possible, which was about 750 participants. The study sample was obtained from 6 different cities in Jordan: Amman, Madaba, Irbid, Karak, Fuhais, and Mafraq. This covered most of the geographic urban areas hosting Syrian refugees. The inclusion criterion was being a Syrian refugee living in Jordan during the study data collection period, aged 18 years or older, and willing to participate in the study. The participants were contacted during their visit to 1 of the 6 Caritas centers from November 2013 through June 2014.

To ensure homogenous data collection and reduce errors, the on-site Caritas workers (psychologists and social workers) were trained to collect data by 1 of the authors. The study aim, objectives, data collection procedure, and inclusion criteria were all explained. Caritas workers contacted each refugee reported to their centers and asked eligible refugees about their willingness to participate in the study, explaining the study objective to them and providing them with the study information sheet. Consent forms were obtained from those who agreed to participate. The demographic data sheet and the questionnaire were distributed to the participants to self-complete in Arabic. Illiterate participants were guided by Caritas workers who read the information to participants and recorded participants’ responses.

Our research is the primary analysis of a large study. Participants’ depression, demographics, chronic diseases, and medication availability were measured. Depression was assessed by using the Beck Depression Inventory (BDI) (9). The BDI has 21 questions to assess depressive disorders in normal and clinical populations. The BDI-II was translated to Arabic by Ghareeb (10) and applied among university students and clinical populations. Previous researchers who used the Arabic version of BDI-II found satisfactory reliability (Cronbach’s α of 0.83) among 19 different Arabic-speaking countries (11).

In our study, the reliability of the BDI-II was checked and had a Cronbach’s α of 0.90, which indicates satisfactory reliability of this scale among the Syrian refugees in Jordan. The data collection package included demographic data in addition to the Arabic version of the revised BDI-II. The total depression scores ranged from 0 to 63. Depression scores were classified as not significant depression (1–30) or significant (31 and above).

SPSS version 21.0 statistical software (IBM, Inc) was used to analyze the data. Descriptive statistics were used to analyze the demographic data. To select the most important factors associated with depression and to include them in the logistic regression in later analysis, a χ^2^ test for independence was used to assess the association between depression categories and other study variables, demographics, previously diagnosed chronic diseases, newly diagnosed chronic diseases, and medication availability. Direct logistic regression was performed to assess the impact of factors that showed significant association with depression on the likelihood that participants would report that they have depression. Significance was set at less than .05.

Ethical approval to conduct the study was obtained from the Caritas Jordan higher management. Written informed consent and information sheets were provided, participation was completely voluntary, and participants had the right to withdraw from the study at any time.

## Results

### Sample characteristics

Of the 811 people asked to participate in the study, 773 accepted and signed the informed consent form (a response rate of 95.3%). However, only 765 were included in the analysis because the remaining participants did not complete most of the data package. Data were collected from the 6 cities, with the highest representation of refugees in Amman (n = 250, 32.7%) and the lowest in Karak (n = 66, 8.6%) (Table 1). Most recruited refugees were aged 18 to 49 years (n = 658, 86.0%), female (n = 425, 55.6%), had resided in Jordan as refugees for less than 1 year (n = 458, 59.9%), were married (n = 656, 85.8%), lived with their family in Jordan (n = 648, 84.7%), had received education in school (n = 668, 87.3%), were unemployed (n = 698, 91.2%), did not have enough income (n = 651, 85.1%), paid house rent (n = 591, 77.2%), did not have a previously diagnosed chronic disease (n = 528, 69.7%), had not been diagnosed with at least 1 chronic disease since arriving in Jordan as diagnosed by 1 of the health care facilities (n = 530, 72.2%), and reported not having enough medications (n = 550, 71.9%) (Table 2).

**Table 1:** Living Location of Syrian Refugees (n = 765) and Association With Depression,^a^ Jordan, 2013–2014

Living Location	No. (%)	No. (%) of Participants With Depression Scores ≤30	No. (%) of Participants With Depression Scores ≥31	χ^2^ *P* Value
Amman	250 (32.7)	180 (72.0)	70 (28.0)	.09
Madaba	97 (12.7)	74 (76.3)	23 (23.7)	
Irbid	112 (14.6)	67 (59.8)	45 (40.2)	
Karak	66 (8.6)	48 (72.7)	18 (27.3)	
Fuhais	102 (13.3)	68 (66.7)	34 (33.3)	
Mafraq	138 (18.0)	102 (73.9)	36 (26.1)	

a As measured with the Beck Depression Inventory (9) translated into Arabic (10).

**Table 2:** Demographics and Study Variables for Depression^a^ Association, Syrian Refugees in Jordan (n = 765), 2013–2014

Factor	Total No. (%)	No. (%) of Participants With Depression Scores ≤30	No. (%) of Participants With Depression Scores ≥31	χ^2^ *P* Value
**Age, y**				
18–49	658 (86.0)	457 (69.5)	201 (30.5)	.08
≥50	107 (14.0)	82 (76.6)	25 (23.4)	
**Sex**				
Male	340 (44.4)	238 (70.0)	102 (30.0)	.81
Female	425 (55.6)	301 (70.8)	124 (29.2)	
**Length of residency in Jordan, y**				
<1	458 (59.9)	314 (68.6)	144 (31.4)	.17
≥1	307 (40.1)	225 (73.3)	82 (26.7)	
**Marital status**				
Single	109 (14.2)	79 (72.5)	30 (27.5)	.65
Married	656 (85.8)	460 (70.1)	196 (29.9)	
**Live with family**				
Yes	648 (84.7)	459 (70.8)	189 (29.2)	.58
No	117 (15.3)	80 (68.4)	37 (31.6)	
**Education level**				
Illiterate	97 (12.7)	73 (75.3)	24 (24.7)	.28
Education in a school	668 (87.3)	466 (69.8)	202 (30.2)	
**Have job**				
Yes	67 (8.8)	52 (77.6)	15 (22.4)	.20
No	698 (91.2)	487 (69.8)	211 (30.2)	
**Have enough income**				
Yes	114 (14.9)	90 (78.9)	24 (21.1)	.03
No	651 (85.1)	449 (69.0)	202 (31.0)	
**Rent house**				
Yes	591 (77.2)	417 (70.6)	174 (29.4)	.93
No	174 (22.8)	122 (70.1)	52 (29.9)	
**Previously diagnosed chronic disease(s)^b^**				
Yes	229 (30.3)	149 (65.1)	80 (34.9)	.04
No	528 (69.7)	384 (72.7)	144 (27.3)	
**Newly diagnosed chronic disease(s) in Jordan^b^**				
Yes	204 (27.8)	123 (60.3)	81 (39.7)	.001
No	530 (72.2)	394 (74.3)	136 (25.7)	
**Have enough medication**				
Yes	215 (28.1)	167 (77.7)	48 (22.3)	.006
No	550 (71.9)	372 (67.6)	178 (32.4)	

a As measured with the Beck Depression Inventory (9) translated into Arabic (10).

b Some data were missing. Analysis of previously diagnosed chronic diseases (s) was based on 757 participants, and analysis of newly diagnosed chronic disease(s) in Jordan was based on 734 participants.

### Depression scores and participant demographics

The depression scores of each BDI-II item were normally distributed. The descriptive results show that 539 (70.5%) of the participants had depression scores of 30 or less (ie, not significant depression), and 226 (29.5%) had depression scores of 31 or more (ie, significant depression).

The descriptive analysis of the depression categories with each demographic variable and the study’s main variables showed that most participants had a depression score of 30 or less independent of their age group, sex, marital status, whether they lived with family members, education level, income, housing status, chronic diseases, and availability of medications (Tables 1 and 2).

A χ^2^ test for independence was used to assess the significance of the association between depression categories, each demographic variable, and the study’s main variables. We found no significant association between depression and participants’ age, sex, duration of stay in Jordan as a refugee, marital status, whether they lived with their family, education level, where they lived in Jordan, and whether they paid house rent. However, the χ^2^ test for independence indicated that there are significant associations between depression categories and participants income (*P* = .03), history of chronic disease before arrival in Jordan (*P* = .04), diagnosis of chronic disease in Jordan (*P* = .001), and availability of medications in Jordan (*P* = .006) (Tables 1 and 2).

Direct logistic regression was performed to assess the impact of factors that showed significant association with depression severity on the likelihood that participants would report that they have depression. The model contained 4 independent variables (income, history of previous chronic disease, newly diagnosed chronic disease in Jordan, and medication availability). The full model containing all predictors was significant, *P* = .001, indicating that the model was able to distinguish whether participants reported depression. The model as a whole explained between 3.1% (Cox and Snell *R* ^2^) and 4.4% (Nagelkerke *R* ^2^) of the variance in depression status, and correctly classified 70.3% of cases. Two out of 4 independent variables made a significant contribution to the model (Table 3): diagnosed with chronic disease in Jordan and medication availability. The strongest predictor to depression was newly diagnosed with chronic disease in Jordan, with an odds ratio of 1.73 that indicated participants with newly diagnosed chronic diseases were more likely to have depression than those who did not have newly diagnosed chronic diseases, controlling for all other factors in the model. The odds ratio of 0.61 for medication availability was less than 1, indicating that with 1 increase in medication availability respondents were 0.61 times less likely to report depression, controlling for other factors in the model.

**Table 3:** Logistic Regression to Predict Likelihood of Reporting Depression Among Syrian Refugees in Jordan, 2013–2014

Variables	β	SE	Wald Test	*df*	*P* Value^a^	Odds Ratio (95% CI)
Income	−0.361	0.255	2.001	1	.16	0.70 (0.42–1.15)
History of chronic disease(s)	0.160	0.197	0.660	1	.42	1.17 (0.08–1.73)
Newly diagnosed with chronic disease(s) in Jordan	0.549	0.200	7.535	1	.006	1.73 (1.17–2.56)
Medication availability	−0.503	0.199	6.370	1	.012	0.61 (0.41–0.89)
Constant	−0.909	0.119	58.169	1	.001	0.403

Abbreviations: β, logistic coefficient; CI, confidence interval; *df*, degrees of freedom; SE, standard error.

a Significance set at *P* < .05.

## Discussion

In this study, although depression was significantly associated with diagnosed chronic diseases in Jordan and medication shortage, the variables studied were not substantial predictors for depression, because the model tested explained less than 5% of the variance in depression status. Also one-third of the study population had depression. Depression was comorbid in 35% of participants with previously diagnosed chronic diseases and in 40% of participants with newly diagnosed chronic diseases.

Since there are no published data related to depression risk and chronic diseases in Syrian refugees, we compared our results with international refugee studies. Reported depression rates in similar studies were inconsistent (11). One study reported depression prevalence of up to 68% (12). However, our results are consistent with recent similar studies. Depression was evident in almost 28% of conflict survivors in Nepal and in 22% of a displaced population in Sri Lanka (13,14).

Chronic disease prevalence in our study is similar to findings of previous studies. In studies of Iraqi refugees, the prevalence of hypertension among refugees in Jordan was 40% (8), and the prevalence of diabetes among refugees in the United States was 35% (15). Similarly, 25% of a group of heterogeneous refugees in the United States reported similar chronic disease rates (16).

In our study, newly diagnosed chronic diseases in Jordan and medication shortages contributed to the study outcome. However, they cannot be considered predictive, as the tested model had a low effect size. Depression in Syrians may be explained by other factors such as trauma, access to food or clean water, family loss, and financial loss, which were not included in our study (17–19).

The spread of chronic diseases among refugees is a challenge because it is associated with illness and death (20). Chronic disease presence in this population may be attributed to premigration or postmigration stressors (21). Many traumatized individuals may have impaired neuroendocrine functioning of the hypothalamus-pituitary-adrenal axis, reflected by cortisol levels (22). Furthermore, contributing factors could be lack of sustainable medical care especially at secondary and tertiary levels (23), lack of medications, absence of patient awareness, and the difficulty of maintaining refugee follow-up in clinics (24).

Clinically, depression may affect chronic conditions such as coronary artery disease (25) and diabetes (26). Although there is a connection between depression and chronic diseases, the underlying pathophysiologic mechanism has to be further studied. Psychological arousal may lead to impairment in neurochemical signaling in the central and peripheral nervous systems, causing subsequent manifestations that are related to cardiovascular functioning and metabolism (27).

Low education levels, low socioeconomic status, unsuitable lifestyle, and lack of medications driven by crisis conditions may exacerbate a patient’s physical health and mood status, thus affecting mental health. Therefore, to alleviate depressive symptoms in Syrian refugees, we recommend adopting effective population-based and pharmaceutical measures to prevent the onset and progression of chronic diseases. Providing patient education programs about chronic diseases and mental illnesses and their risk factors, emphasizing the importance of attending routine check-ups for chronic diseases, and encouraging adherence to therapy are useful methods.

One of our study’s main strengths was the large sample size. Participants were recruited from different cities, and illiterate and literate participants were included, thus ensuring reliable representation and accuracy. Moreover, the study used a reliable and valid instrument to assess depression. On the other hand, the study had some limitations. Because entry to refugee camps is prohibited, data were collected only from Syrian refugees in urban areas. The data were self-reported, thus leading to potentially inaccurate data. Another limitation was in chronic disease records. Participants were asked if they had chronic diseases in general without specifying chronic disease type or reporting comorbidity with other chronic diseases. Moreover, chronic diseases were not diagnosed or confirmed clinically. Also it is unknown for how long patients lacked medications. The study did not focus on other physical health issues and disabilities such as pain or musculoskeletal disorders that may contribute to refugees’ mental health. Finally, this study focused only on depression as an important mental disorder in Syrian refugees residing in Jordan; however, posttraumatic stress disorder remains important to be studied in future.

On the basis of the above results, we recommend early screening and identification and management of priority chronic diseases among Syrians as soon as they enter Jordan. Also, health care professionals should take a more active role in patient education. We recommend that further studies include additional factors that may predict depression among Syrian refuges in Jordan. Prompt measures have to be taken to better identify and prevent the spread of chronic diseases and maintain the mental health of this fragile population.
